# Methodology for development of a data and knowledge base for learning from existing nature-based solutions in Europe: The CONNECTING Nature project

**DOI:** 10.1016/j.mex.2020.101096

**Published:** 2020-10-13

**Authors:** Diana Dushkova, Dagmar Haase

**Affiliations:** Department of Geography, Humboldt University Berlin, Germany

**Keywords:** Nature-based solutions (NBS), Data- and knowledge base, Climate change, Societal challenges, Sustainability, Resilience, Urban Europe

## Abstract

Within CONNECTING Nature, we are dealing with developing innovative nature-based solutions (NBS) for climate change adaptation, health and well-being, social cohesion and sustainable economic development in European cities. In order to enable “learning by comparing” and “generating new knowledge” from multiple NBS related studies, a novel data and knowledge base is needed which requires a specified methodological approach for its development. This paper provides conceptual and methodological context and techniques for constructing such a data and knowledge base that will systematically support the process of NBS monitoring and assessment:

• A methodology presents the comprehensive, multi-step approach to the NBS data and knowledge development that helps to guide work and influence the quality of an information included.
• The paper describes the methodology and main steps/phases for developing a large data and knowledge base of NBS that will allow further systematic review.
• The suggested methodology explains how to build NBS related databases from the conceptualization and requirements phases through to implementation and maintenance. In this regard, such a methodology is iterative, with extensive NBS stakeholders’ and end-user's involvement that are packaged with reusable templates or deliverables offering a good opportunity for success when used by practitioners and other end-users.
• The NBS data and knowledge base gathers information about different NBS models and generations into one easy-to-find, easy-to-use place and provides detailed descriptions of each of the 1490 NBS cases from urban centers in Europe.
• The data and knowledge base thus helps users identify the best and most appropriated NBS model/type for addressing the particular goals and, at the same time, considers the local context and potential.
• The data obtained can be used for the further meta-analysis by applying statistics or searching for specific sample cases and thus enables to generate and expand the knowledge from multiple NBS related studies, in both qualitative and quantitative ways.

## Graphical abstract

**Figure:**
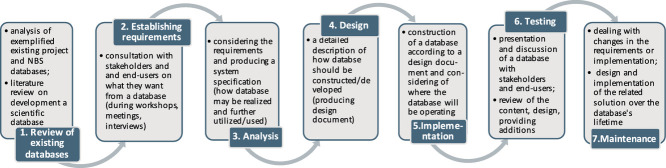

Specifications table

**Table:** 

Subject Area:	Environmental Science
More specific subject area:	*Environmental Science* *Data mining*
Method name:	*There is no specific method that was originally developed and then modified. We used different methods from NBS related projects and platforms to develop a tool for value and data extraction.*
Name and reference of original method:	*Original sources include:* a) *database systems and database design concepts were derived from:* • A. Watt, N. Eng, Database Design. 2nd ed. BCcampus, 2014. https://opentextbc.ca/dbdesign01/ • S.E. Hampton, C.A. Strasser, J.J. Tewksbury et al., Big data and the future of ecology. Front Ecol Environ 11(3) (2013) 156–162, doi:10.1890/120,103 • V.B. Chaudhary, L.L. Walters, J.D. Bever, et al., Advancing synthetic ecology: a database system to facilitate complex ecological meta-analyses. Bull Ecol Soc Amer 91 (2010) 235–43. *b) Review of approaches to NBS database development from:* • Naturvation: Urban Nature Atlas – a Database of Nature-Based Solutions Across 100 European Cities. https://naturvation.eu/atlas • Oppla – the EU Repository of Nature-Based Solutions. Available online: https://oppla.eu/case-study-finder • ThinkNature: Platform for Nature-Based Solutions. https://www.think-nature.eu/
Resource availability:	*The data- and knowledge base is currently in the process of being made publicly available and accessible online for a broad range of stakeholders, actors and researchers interested in the creation, development and implementation of NSB. The data and knowledge base was originally developed in Microsoft Excel and is currently in the process of being published on the CONNECTING Nature website. It will be freely accessible at*https://connectingnature.eu/*.*

## Background

The concept of nature-based solutions (NBS) is increasingly recognized for its potential to address the current societal challenges in cities that have resulted from (and are amplified by) climate change, environmental pollution, depletion of natural resources, loss of biodiversity and decreasing quality of life and well-being [1], [2], [3], [4]. By incorporating the main ideas of green and blue infrastructure (GBI), ecosystem services (ES) and biomimicry concepts, the creation and development of NBS is considered to be a new urban design strategy and planning tool for the building of resilient, sustainable, livable and healthy cities [5], [6], [7], [8]. As underlined by EC [9] and EU [8], research and innovation on NBS should include novel approaches, while also building on existing tools, methods, knowledge and data and knowledge bases. In particular, one of the core principles and additional strategies that is recommended for the creation and implementation of NBS in cities is connecting existing networks as well as “learning by comparing'' [9]. This was one of the aims of the EU funded Horizon 2020 project CONNECTING Nature (www.connectingnature.com).

In order to map and build-on existing knowledge, and innovate when creating and implementing a new generation of NBSs, it is essential to generate substantial new knowledge in terms of data (e.g. reference data) and processes (e.g. for large scale deployment of NBS in urban contexts). It requires a specified methodological approach adopted when dealing with NBS research and innovation. Thus, the aim of this paper is to provide both methodology and techniques for design and implementation such a data and knowledge base that will systematically support the process of NBS monitoring and assessment. It describes main steps and phases for developing systematic knowledge about NBS that will allow further review and analysis. In particular, the suggested methodology explains how to build NBS related databases from the conceptualization and requirements phases through to implementation and maintenance. In this regard, such a methodology is iterative, with extensive NBS stakeholders’ and end-user's involvement that are packaged with reusable templates or deliverables offering a good opportunity for success. This easy-to-use and replicate methodology has a structured approach, provides phases to guide in the design process and assist the users to:

• highlight the importance and relevance of NBS for sustainable and livable cities and urban regions,
• elaborate on a data and knowledge base where the efficiency, effectiveness and sustainability of NBS interventions can be systematically assessed and analyzed,
• reveal a range of indicators that highlight and address the connections between NBS and other goals of sustainable and climate-proof urban development (e.g. creation of resilient cities, adaptation to climate change, and the promotion of the health and wealth of urban residents), and finally,
• gain a new sort of knowledge based on the analysis of specific NBS projects and their profile across European cities.

### Why is there a need for a new methodical approach to development of such an NBS-related data and knowledge base?

There are several advantages in developing and using scientific data and knowledge bases. First, data and knowledge bases lead to an overall improvement in data quality. The rise in user numbers increases the frequency of the detection and correction of problems in the data. A second advantage is that data and knowledge bases are cost-effective and allow the saving/storing of important information in a categorized form — even when the project is finished and the source or original information (e.g. website) is no longer available. As a computerized, organized collection of related data that can be accessed for theoretical and practical inquiry and long-term stewardship, scientific data and knowledge bases allow the integration of dissimilar data sets and generate a new sort of knowledge by applying a new analysis approach that is often multi- and interdisciplinary in nature. Although there are several projects and platforms that present NBS data, these datasets are widely scattered, have different data formats and conventions, and their accessibility is often limited. Moreover, such datasets originate from different original sources and often do not provide the desired spatial and temporal resolutions. The information about NBS and the benefits provided by them is scattered across websites (often established in the local languages and thus not accessible to the broader public), government reports, journals. Sometimes the information is no longer accessible after the period of NBS implementation is over and when no monitoring is undertaken. In some cases, the provided original websites do not exist anymore making it potentially difficult for a user to locate the desired information regarding a particular NBS case or a specific NBS category or model.

Within the CONNECTING Nature project, we endeavored to elaborate a specified methodological approach which will be appropriate when dealing with creation of NBS related databases. In doing so, a concept and methodology was developed which provides description of techniques for design and implementation such a data and knowledge base. The particular need for creation of such methodology lies in the field that in comparison with other methodologies of database creation, methodology of NBS data and knowledge base requires the reflection of the following criteria:

• it systematically supports the process of NBS implementation, monitoring and assessment;
• it describes main steps/phases for developing a large data and knowledge base of NBS that will allow further systematic review;
• the suggested methodology explains how to build NBS related databases from the conceptualization and requirements phases through to implementation and maintenance. In this regard, such a methodology is iterative, with extensive NBS stakeholders’ and end-user's involvement that are packaged with reusable templates or deliverables offering a good opportunity for success;
• this easy-to-use and replicate methodology has a structured approach and provides phases to guide in the design process and assist the users.

The method described below uses data on different NBS models and generations into one easy-to-find, easy-to-use place and provides detailed descriptions for each of the 1490 NBS cases from urban Europe. This helps users identify the best and most appropriated NBS model/type in order to address the particular SDG and, at the same time, to consider both local context and potential. End users (stakeholders, scientists, different urban actors dealing with NBS) can compare NBS motivations (objectives), the environmental contexts where they were realized, and societal challenges aimed at addressing their feasibility in light of their specific search and needs.

Considering the lessons learned from the establishment of related NBS datasets and reflecting the feedback from the CONNECTING Nature project partners (e.g. different stakeholders and scientists from government, academia and business), we developed a methodology for creation of a data and knowledge base to provide sustainable support for innovation, exploitation and enterprise development when dealing with NBS. In this regard, the current version of the NBS data and knowledge base developed in CONNECTING Nature can be seen as an element of a Europe-wide, comprehensive and robust mechanism that will be used to monitor and evaluate the effectiveness of NBS implementation in cities, firstly, with regards to the database specific impact categories (flooding, heat, health etc.), and secondly, in terms of fulfilling several criteria of long-term deployment and sustainability.

The methodology for creation of NBS data and knowledge base developed within the CONNECTING Nature project provides essential information on how to allow different users to systematically assess and analyze the efficiency, effectiveness and sustainability of urban NBS interventions in Europe. In our earlier publications, we presented a series of first lessons that were extracted from the data and knowledge base for the development of an impact assessment framework of NBS in Europe [10], [11], [12], [13]. In this MethodsX paper, we methodologically describe the major steps/phases, challenges, and considerations of building an integrated data and knowledge base of nature-based solutions as an online platform for finding, examining and comparing different categories and models of existing nature-based solutions (NBS). It will serve the creation, development and implementation of future NBS or NBS datasets that aim to address the same sustainable development goals (SDGs) [14] or are derived from similar motivations and expected benefits.

## Materials used to create a data and knowledge base

The information sources for creation such a NBS related data and knowledge base can be obtained from the existing online databases or large international and national projects dealing with NBS and GBI in cities. In the case of the CONNECTING Nature data and knowledge base, the data sources were identified by the Consortium of CONNECTING Nature project and listed in previous documents as the main sources (such as OPPLA, iSCAPE, GLAMURS, GREENSURGE, ARTS, GUST, AMICA, IMPRESSIONS, OPERAs, PLUREL, URBACT, SUSTAIN, TRANSIT, TURAS, URBES etc.; see Table 1 of Supplementary Material).

Each NBS case included in our NBS related data and knowledge base was primarly based on secondary sources (e.g. project reports, deliverables and other project documents, websites, news articles, blogs entries etc.). For each NBS case, we provided information sources and contact persons. In some cases, where information on NBS was limited, personal communication with the study coordinators and individuals involved in the projects was performed via email and phone calls, as well through the use of short questionnaire surveys (as primary data). This has provided further and more specific information about the NBS initiatives, and has helped to identify key points for this work. Additional literature that was used to find respective case studies includes scientific papers and reports of different European agencies concerned with implementation of particular NBS, the environment and climate change, reports of adaptation platforms, book chapters, government documents, and reports by the stakeholders involved in the project.

## Method details

### Methodical approach for the development of the NBS data and knowledge base

**Fig. 1:**
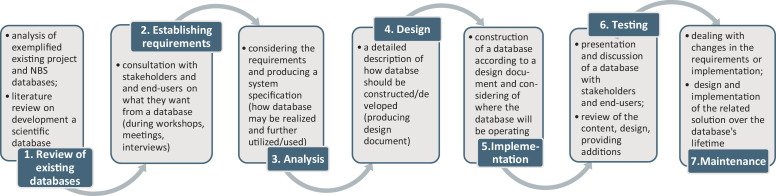
Overview of the NBS data and knowledge base development life circle created within CONNECTING Nature project.

The current version of the CONNECTING Nature data and knowledge base was developed between June 2017 and May 2020. Methodically, the model illustrating the scope of activities that took place during the data and knowledge base creation, development and implementation process is shown in Fig. 1. A core aspect of this methodical approach is the subdivision of the development process into a series of steps or phases, each of which focuses on one aspect of the development. The collection of these steps referred to so-called NBS database life cycle. Each phase in the life cycle can be checked for correctness before moving on to the next phase. As a *first step* in development of a data and knowledge base we identified a review analysis based on examples of existing NBS data and knowledge bases and related literature review. This was carried out between June and October 2017 and based on those developed by Naturvation (https://naturvation.eu/home), OPPLA (https://oppla.eu/case-study-finder), or Think Nature (https://platform.think-nature.eu/). We also conducted a literature review of published papers, books and guidelines on how to develop a scientific data and knowledge base [15], [16], [17], [18], [19], [20]. Considering the data-intensive nature of socio-ecological systems presented by NBS, we also suggest to apply here the methods developed by Recknagel and Michener [21] on how to create a big data and knowledge base where valuable information can be found in a wide range of ecological, social, economic data. This information can also be used to address the need to communicate results and inform decision makers of the research and implementation of NBS. The *second step* refers to establishing requirements which involves consultation with, and agreement among, stakeholders about what they want from this database, expressed as a statement of requirements. For this reason, between September 2017 and November 2017, the first draft of the data and knowledge base concept (structure and attributes) was developed and discussed with the responsible project teams (UDC and DRIFT). The draft was also presented to the project partners including the main end users from the government, academia and SMEs. The *third step* – Analysis – starts by considering the statement of requirements and producing a system specification, in other words: how database may be realized and further utilized/used. The *fourth step* relates to design of a data and knowledge base which begins with a system specification and include producing design documents and a detailed description of how a system should be constructed. In this regard, between October and November 2017, the concept of the data and knowledge base was finalized and its structure were re-designed to take the discussion on the project workshop and feedback from the project partners into consideration. At this point, we began collecting data (*step 5* – Implementation). This *fifth step* includes the construction of a computer system according to a desired design document and should consider the context in which the data and knowledge base will be operating (e.g., specific software available for the development). Implementation should be accompanied with a testing (*step 6*) which help to reveal how appropriate is the released design and content for use. During the whole development process of the data and knowledge base, we participated in a myriad of discussions and consultations with project partners, local stakeholders and end-users. These discussions resulted in a review of the content and design and also expanded the content by providing additional information.. Since we propose a methodical approach for creation a data and knowledge base as a living document, we find the *seventh step* – maintenance – as an important step which helps to deal with changes in the requirements or implementation, design and implementation of the related solution over the database's lifetime.

The current data and knowledge base includes 1490 cases and is handled as a ‘living document’ across the project lifetime. It is intended to be finalized and stored at the end of CONNECTING Nature. Accordingly, all NBS projects followed in the CONNECTING Nature Frontrunner and Fast Follower cities are incorporated in the data and knowledge base and can serve as learning material and as a ‘living document platform’ and thus will be continuously maintained and update (*step 7).* A ‘living’ version of the CONNECTING Nature NBS data and knowledge base (it is an object of continual change/modification by the active and regular addition of new data) is also being regularly updated at the CONNECTING Nature project webpage. A version with suitable NBS cases was developed for non-academic/non-expert audiences and sent to the City Council of each participating city in CONNECTING Nature.

The data and knowledge base has been established in MS Excel format and can be easily transferred into other statistics software via the .txt or .csv exchange formats. The main criteria for including a NBS project in the data and knowledge base are: the project being located in a European city and whether or not the project addresses social-environmental issues or challenges such as sustainable development goals listed by the UN (2015). A first data analysis of the initial 343 NBS was carried out between November 2017 and February 2018. This was done in order to assess the data and present the findings in graphic form and to better visualize the data during several FRC workshops in 2018. From this time onwards, the data and knowledge base was extended and now contains 1490 urban NBS interventions from 32 countries and 235 cities.

### Content

**Fig. 2:**
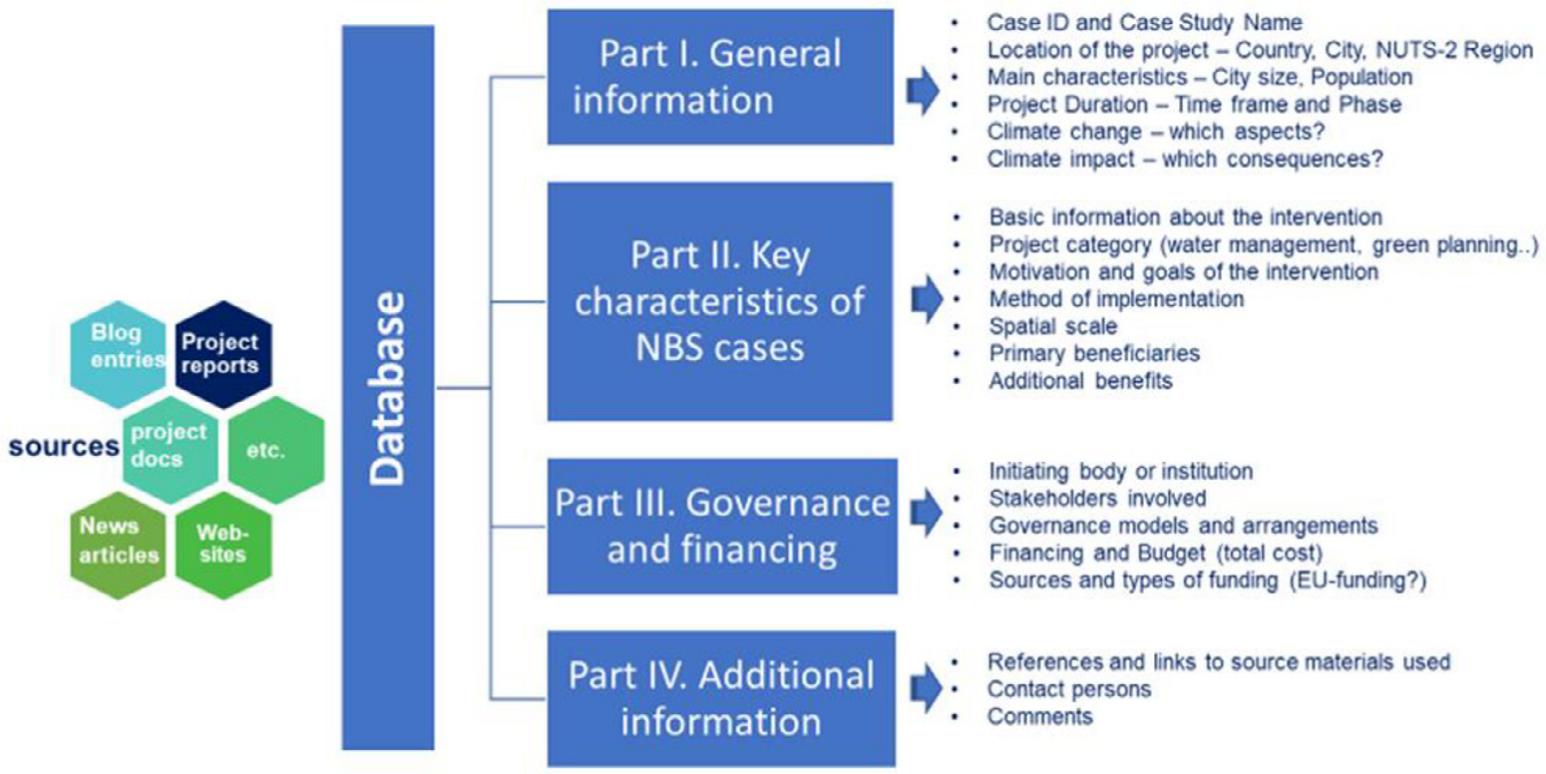
Information sections presented in the CONNECTING Nature data and knowledge base.

The NBS data and knowledge base is equipped with search functionality that simplifies the process of data gathering for users. The data and knowledge base does not follow a spatial mapping logic and does not seek for any European/national representation (such as a minimum threshold number of NBS per city, region or country). It is due to this fact that distribution of NBS documentation is not equally represented (having lower number of claimed NBS cases for Eastern Europe and the most often appearing NBS cases from Central and Western Europe) The main search function follows a thematic logic of searching by key characteristics (Fig. 2), categories and variables (Table 2 of Supplementary Material), however, a short description of each NBS case was also provided.

A large number of European cities are poorly represented in NBS research, innovation and demonstration programs, which have often focused on particular areas. In addition, while the majority of European urban zones are either small or medium sized, there are many large cities that are also in need of a large-scale response to the challenges of climate change, rising temperatures, occasional and regional flooding, or severe water stress. Thus, it was decided that NBS cases presented in the data and knowledge base cover a broad range of city sizes ranging from small (urban population of 50,000–100,000), via medium (100,000–250,000), to large (250,000–500,000+), extra-large (500,000–1000,000) and finally XXL (1000,000–5000,000) cities that were respectively categorized. The NBS included in the CONNECTING Nature data and knowledge base were also chosen according to their ability to accommodate the challenges that cities manifest. The NBS were also chose for their potential impact on the ability of these cities to build resilience, including social aspects such as public health, human wellbeing (especially the financial implications of both), and also cultural heritage, unemployment and migration.

The information included in this data and knowledge base covers the key characteristics of urban NBS, governance arrangements that enable their implementation, direct beneficiaries and impacts, the type of innovation of different NBS domains, among others. Details are presented in Fig. 2.

As shown in the Fig. 2, the CONNECTING Nature data and knowledge base consists of four parts:

*Part I*, general information, presents location and description of the project in which basic information about the NBS intervention is requested, such as the Case ID and Case study name, country, NUTS-2 region, and city of origin with city size (population). It also provides the time frame and phase of the NBS interventions (finalized, ongoing, in operation, pilots ongoing, design progress (submitted). This section also indicates if the intervention is a NBS, partly a NBS, or even non-NBS (meaning that it is a project that deals with gray infrastructure). This section also shows if the NBS relates to climate change (yes, no). It includes the specific aspects of climate impact (which consequences) by indicating the following categories: flooding, heat and drought, loss of biodiversity, air quality, not relevant, education purpose, and energy consumption.

*Part II*, key characteristics of the NBS cases present basic information about the NBS intervention (goals of the intervention are given as a short description of the individual NBS), project category (big data, governance and planning, changing mentalities, workshop, gray infrastructure, citizen engagement, citizen and stakeholder engagement). These key characteristics also underline motivation – the following categories were presented: climate change adaptation and mitigation, quality of life/human well-being/public health/attractiveness of place, nature conservation, water management and flood control, improved planning, awareness raising, sharing knowledge, citizen engagement, producing new knowledge, citizens and stakeholder engagement, and networking. Also, information on method of implementation is provided such as: change in physical infrastructure, change in legislation and regulation, financial incentives, methods for integration ESS, big data, networking, sharing knowledge, application of best-practice methods, green infrastructure development. This section also identifies the spatial scale (continental, national, regional, city, city district, site) and primary beneficiaries (public, private, public-private, governmental institutions, scientific or technical advisors). It also underlines additional benefits such as: climate change mitigation and adaptation, social inclusion, improved (green) planning, big data, awareness raising, citizens and stakeholder engagement, sharing knowledge, networking, sustainable living, different, and no additional benefits measurable.

*Part III*, governance and financing, provides information on governance arrangements, including initiators (public, private, public-private, governmental institutions, scientific or technical advisors), key actors and stakeholders (public, private, public-private, governmental institutions, scientific or technical advisors) involved in the planning and implementation of NBS. It also includes financing aspects such as the sources of funding, total cost and types of funding used (also by indication of EU sources).

*Part IV*. Additional information provides references and links to source materials used and provides data on contact person(s).

## Classification scheme, categories and key variables

As the process of organizing data by relevant categories, data classification enables users to use data more efficiently by making the data easier to locate and retrieve. In our case, data classification involved tagging data to make it easily searchable and trackable. The data classification process, as well as the key classification categories and variables, were discussed with the end-users in order to check whether such a classification scheme corresponds to their needs and the usability/application aspects. In summary, the main goal of our classification scheme was to ensure that NBS related data can be used for a variety of purposes, are easy to access, maintain regulatory compliance, and meet various other users’ objectives.

**Fig. 3:**
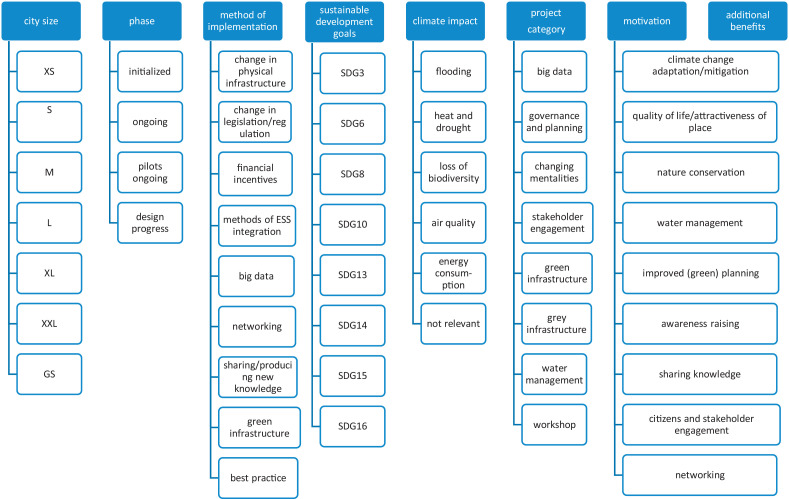
Scheme presenting key characteristics of Part II of the data and knowledge base.

**Fig. 4:**
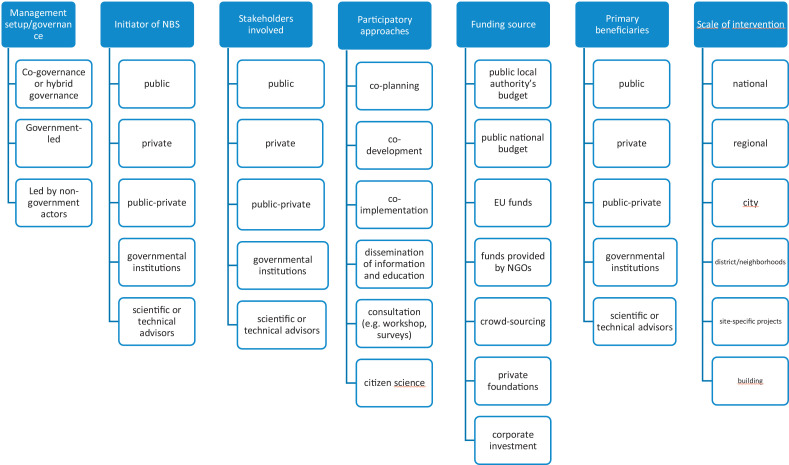
Key characteristics and their variables included in the Part III (Governance and financing).
○ The key variables of the generated categories (see their description in detail presented in Table 3 of Supplementary Material) can be systematically analyzed later using frequency distributions, spider diagrams, correlation and qualitative clusters.

In order to prepare the data for the further analysis and meta-analysis, we undertook the following working steps:

○ First, we categorized the NBS interventions in different classes according to their scale and scope (Table 2 of Supplementary Material).
○ We applied different categories and corresponding key variables (Figs. 3 and 4) to extract data from the case study information in order to approach the research questions in the following statistical analysis.

Part I and Part IV include the information that is not categorized and thus was not represented by variables (e.g. case ID and name of NBS, population size, NUTS2 region, country, city, short project description, information source, and contact person).

**Fig. 5:**
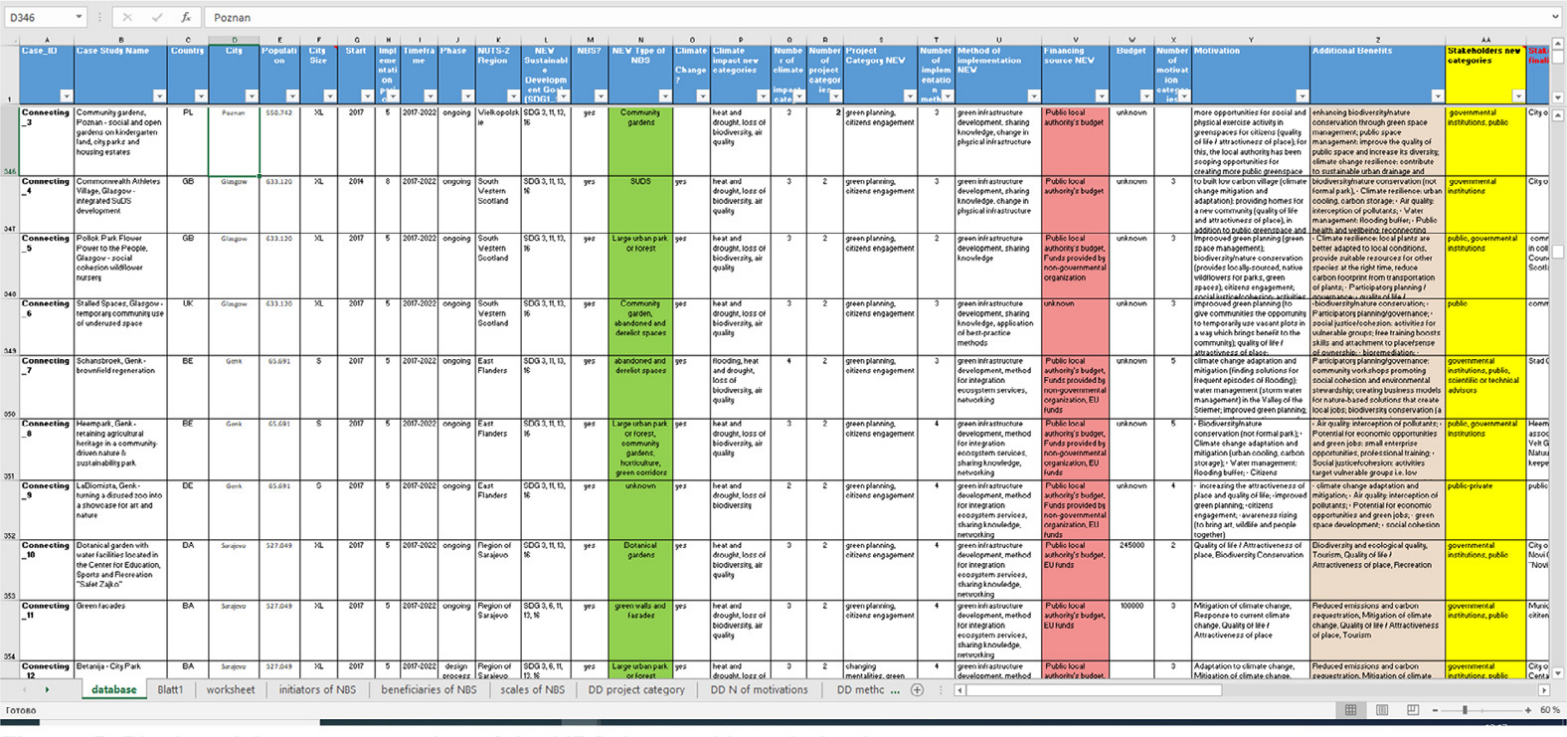
Display of the current version of the NBS data and knowledge base.

The data collected has also been transferred to an Excel data and knowledge base which allows for more detailed statistical analysis of the entire data sample based on selected key variables, features and characteristics of the NBS projects (Fig. 5). In the data and knowledge base, each row starts with a one-to-one ID and contains all data provided for a specific NBS intervention (case). Each column displays the information provided for a specific category, key variable or feature.

A display snapshot (Fig. 5) of the NBS data and knowledge base shows the criteria columns where different NBS cases are presented according to the main categories and variables through which the data on NBS initiatives have been coded. Structured along the scope or scale of interventions, the key types of NBS-related initiatives are presented in Table 3 of Supplementary Material.

## Data access

The data and knowledge base of CONNECTING Nature can be publicly accessed via the project website (www.connectingnature.eu) after the project life cycle will have ended. The data and knowledge base is cost free and open to be used and integrated with other data. It will be provided in an easy to transfer format such as csv or related transportable formats. In addition, the data and knowledge base will be readable for any user by this article published and provided.

Clear benefits for the user are the concept to structure knowledge about urban NBS, the opportunity to compare data and methods of implementation of NBS across European countries as well as analyzing success and failure factors of existing NBS to avoid the replication of the latter when implementing new NBS. In addition, any user can extend the data and knowledge base when adding own data and cases to this open document.

## Limitations and outlook (further steps in NBS data and knowledge base development as a platform)

Our procedures make a large, complex, and integrated data and knowledge base of different NBS-related data reproducible and extensible. This allows users to ask new research questions with the existing data and knowledge base or through the addition of new data. The largest challenge of this task was the heterogeneity of the data, formats, and metadata since, as was already mentioned, not every NBS project platform or source presented data that included the same variables/characteristics. Thus, there are some steps of data integration that need manual input from experts in diverse fields and that require cross-checking and additional close collaboration.

Review of NBS evaluation frameworks developed within the CONNECTING Nature project as well as NBS cases from our data and knowledge base has revealed a series of conceptual and empirical gaps that should be addressed in order to develop a solid and usable evidence base in the following years and to inform policy decisions in global urban projects. Based on this foundation, we outline further requirements in NBS data and knowledge base development:

• the identification of new synergistic data-gathering techniques that make use of the latest available technologies and make the data available to a broad audience, especially traditionally under-represented groups in urban policy-making;
• since we revealed that NBS projects would benefit greatly from rich and mixed evaluation methods to illuminate their performance on multiple impacts and to identify the synergies and trade-offs between them, it is foreseen to extend the NBS data and knowledge base through manual input from experts in diverse fields;
• cross-case comparability of NBS presented in the data and knowledge base will allow for better extraction of NBS related data which can be further transformed into decision-making tools to support cities and all actors involved in NBS development and implementation processes aim at making the urban environment more social, environmentally friendly and cost-effective.
• there is a need for the innovative design of NBS data platforms where the data and knowledge base will be presented to ensure that a balance can be achieved between rich descriptions of cases on the one hand, and comparability and transferability of results, on the other.

Being aware that the development of a data and knowledge base is an evolutionary process, we do hope that a NBS data and knowledge base such as this will serve a dynamic community of users during its lifetime and will be continuously actualized to meet the changing requirements posed by end-users.

## Conclusions

We presented a methodology for development of the NBS related data and knowledge base as a comprehensive, multi-step approach that helps to guide work and influence the quality of an information included in such a database. We have described the major steps, challenges, and considerations in building an integrated data and knowledge base of NBS that has been developed within the CONNECTING Nature project. This data and knowledge base presents 1490 NBS interventions from 32 European countries and 235 cities. The suggested methodological approach presents a procedure for the development of datasets, that includes: creating a flexible data and knowledge base design; integrating metadata; quality control of integrated and obtained data (e.g. via discussion with end-users), and extensive documentation of the data and knowledge base. Our procedures make a large, complex, and integrated data and knowledge base of different categories of NBS reproducible and extensible. This allows users to ask new research questions with the existing data and knowledge base or through the addition of new data. The largest challenge of this task was the heterogeneity of the data, formats, and metadata. Many steps of data integration require manual input from experts in diverse fields and this entails close collaboration.

Therefore, based on this methodological approach to the NBS data and knowledge base development, the efficiency, effectiveness and sustainability of NBS interventions can be systematically assessed and analyzed using a range of indicators. All this highlights and addresses the connections between NBS and other goals of sustainable and climate proof urban development – e.g. the creation of resilient cities, adaptation to climate change and the promotion of the health and wealth of urban residents. Altogether, these steps finally contribute to the production of a new sort of knowledge based on the analysis of specific NBS projects and their profile across European cities.

## Declaration of Competing Interest

Authors declare no conflict of interest.

## Appendix Supplementary materials

Image, application 1
