# Supplementary material for: Methodology for development of a data and knowledge base for learning from existing nature-based solutions in Europe: The CONNECTING Nature project
Source: MethodsX. 2020 Oct 13;7:101096. doi: 10.1016/j.mex.2020.101096 (PMC7600359; doi:10.1016/j.mex.2020.101096)
Supplement: Supplementary file 1 [file mmc1.docx]

**Supplementary material *and/or* Additional information:**

**Table 1.** Information source for NBS data and knowledge base

| **Project data and knowledge base (source)** | **Description** |
| --- | --- |
| **GraBS** – Green and Blue Space Adaptation for Urban Areas and Eco Towns (www.grabs-eu.org) | Case study database of 15 green and blue adaptation projects for urban areas and eco towns. The aim is to ensure that existing and new mixed-use urban development is adapted to the impact of climate change through the improvement of planning policy, using GBI. |
| **CLIMATE-ADAPT** – European Climate Adaptation Platform http://climate-adapt.eea.europa.eu/ | A partnership between the European Commission and the European Environment Agency (EEA) that provides a comprehensive set of 108 adaptation case studies and adaptation information across Europe. Database contains quality checked information annotated by climate adaptation experts. |
| **The Future Cities Partnership**  www.future-cities.eu | The partnership develops innovative concepts and implementation strategies for cities. The partners cooperate to develop, apply and improve assessment criteria for climate-proof cities. Appropriate action plans are developed in each city and priority measures are implemented. |
| **Mayors adapt**  http://mayors-adapt.eu/ | An initiative of the European Commission, launched in the context of the EU Adaptation Strategy, implemented within the Covenant of Mayors, and supported by the EEA. It provides information about climate change-related actions in the participating cities and shows their phase of implementation. |
| **Green Surge**  www.greensurge.eu | This project aims to identify, develop and test ways of linking green spaces, biodiversity, people and the green economy in order to meet the major urban challenges related to land use conflicts, climate change adaptation, demographic changes, and human health and wellbeing. |
| **OPPLA** – the EU Repository of Nature-Based Solutions  https://oppla.eu | Oppla provides a knowledge marketplace, where the latest thinking on natural capital, ES and NBS is brought together. The goal is to simplify how to share, obtain and create knowledge to better manage our environment. Oppla is an open platform designed for different users. |
| **iSCAPE** (Improving the Smart Control of Air Pollution in Europe)  https://www.iscapeproject.eu | Thid project is concerned with integrating and advancing the control of air quality and carbon emissions in European cities in the context of climate change and through the development of sustainable and passive air pollution remediation strategies, policy interventions, and behavioral change initiatives. |
| **GLAMURS** (Green Lifestyles, Alternative Models and Upscaling Regional Sustainability)  http://glamurs.eu | This project demonstrates strategies and mechanisms for policy making aimed at transitioning to sustainable urban lifestyles that are low-carbon, time-affluent, and rich in wellbeing, as well as opportunities for transitioning to a green economy. Also using knowledge co-production, GLAMURS has developed pathways to show the necessity of healthy lifestyles and the factors influencing them. |
| **ARTS** (Accelerating and Rescaling Transitions to Sustainability)  http://acceleratingtransitions.eu | ARTS is committed to understanding the role and impact of transition initiatives in cities by examining the conditions that can accelerate change towards a sustainable low-carbon society. Advancing transition theory through an evidence-based approach on understanding dynamics of sustainable solutions in cities, it has produced an evidence-based framework for co-creation and social learning. |
| **GUST** (Governance of urban sustainability transitions: advancing the role of urban living labs) www.urbanlivinglabs.net | GUST provides valuable knowledge on Urban Living Labs (ULL), but also exemplifies dissemination and implementation of the project results. It examined governance issues with respect to transitioning strategies in ULL. GUST enabled a synthesis of empirical results and conceptual links between design, practices and processes as well as co-production of knowledge. |
| **AMICA** (Adaptation and Mitigation – an Integrated Climate Policy Approach)  http://www.amica-climate.net | AMICA is a new approach to environmental policy that combines long-term climate protection with short- and mid-term adaptation measures on the local level as a means of improving coherence of decisions and allocation of financial means. The project developed strategies for responding to climate change and reducing vulnerability through exchanging experiences and capacity building. |
| **IMPRESSIONS** (Impacts and risks from high-end scenarios: strategies for innovative solutions)  http://www.impressions-project.eu/ | This project aims to advance understanding of the implications of high-end climate change and to help decision-makers apply such knowledge within integrated adaptation and mitigation strategies. It aims to design a stakeholder-led methodology, that is the conceptual guiding basis of multi-stakeholder workshops, and implement an adaptive governance framework. |
| **OPERAs**  http://www.operas-project.eu | This is the FP7 ecosystem service project which created OPPLA – a global nature-based solution platform. It aims to put cutting edge ecosystem science into practice. Researchers and practioners from 27 different organizations helped stakeholders to put ES and natural capital concepts into practice, devising methods for valuing cultural ES. |
| **PLUREL** (Peri-urban Land Use Relationships – Strategies and Sustainability Assessment Tools for Urban-Rural Linkages) www.plurel.net | This project used multidisciplinary methods to develop four scenarios for Europe's outlook in 2025 as the basis for the study of possible future land use changes in European urban areas in general and, in particular, for six case study regions. It tackled the complex interface between the city edge and its rural hinterland. It developed new strategies and planning and forecasting tools essential for developing sustainable rural-urban land use relationships, evaluated costs for their implementation. |
| **URBACT** (European exchange and learning program promoting sustainable urban development)  https://urbact.eu | The URBACT-funded RESILIENT EUROPE project provides a base upon which to base the policy, planning and local businesses conditions that are required for NBS to scale up. URBACT helps cities to develop pragmatic solutions that are new and sustainable and that integrate economic, environmental, and social urban topics. It aims to foster sustainable urban development through networking and co-learning. |
| **SUSTAIN**  http://www.sustain-europe.eu | A capacity building project aimed at building knowledge and skills in urban sustainable development for young professionals or, simply put, to equip the next generation of urban planners through the use of advanced interactive curricula. The lessons learned include how to connect science to policy and planning capacity building when scaling up to a wider cohort of urban planners and managers. |
| **TRANSIT** (Transformative Social Innovation Theory) http://www.transitsocialinnovation.eu | Lessons from TRANSIT include methods and pathways for developing transformative social innovation as well as social entrepreneurship and crowd funding. This project developed knowledge on how to sustain social innovation networks over time, how to tap into their evolution at local and global scales, and what governance practices exist to effectively stimulate these networks in innovating with NBS. |
| **TURAS** (Transitioning towards Urban Resilience and Sustainability) www.turas-cities.org | This project aims to bring together urban communities, researchers, local authorities and SMEs to research, develop, demonstrate and disseminate transition strategies and scenarios that will enable European cities and their rural interfaces to build vitally-needed resilience in the face of significant sustainability challenges. TURAS devised and demonstrated complex transition strategies through NBS and used the results to create collaborative planning and capacity-building companies. |
| **URBES** (Urban Biodiversity and Ecosystem Services) BiodivERsA  https://www.biodiversa.org/121 | This project addressed significant scientific knowledge gaps on the role of urban biodiversity and ES for human well-being in order to improve the ability of European cities to adapt to climate change and reduce their ecological footprints. It focused particularly on functional diversity, urban ES, economics, and resilience science. It developed a tool box to promote sustainable management of urban biodiversity and ES. |
| **OpenNESS** (Operationalization of Natural Capital and Ecosystem Services) www.openness-project.eu | OpenNESS aims to translate the concepts of Natural Capital and ES into operational frameworks that provide tested, practical and tailored solutions for integrating ES into land, water and urban management and decision-making. It examines how the concepts link to, and support, wider EU economic, social and environmental policy initiatives and the potential. |
| **OPERANDUM*** (OPEn-air laboRAtories for Nature baseD solUtions) www.site.unibo.it | This project develops nature-based solutions (NBS) to mitigate the impact of hydro-meteorological phenomena in risk-prone areas in order to manage environmental risks. It delivers the tools and methods for the validation of NBS and to enhance resilience in European rural and natural areas. |
| **PHUSICOS*** (in Greek φυσικός, meaning 'according to nature')  www.phusicos.eu | PHUSICOS demonstrates how NBS reduce the risk of extreme weather events in rural mountain landscapes. The focus is on demonstrating the effectiveness of NBS and their ability to reduce the impacts from small, frequent events (extensive risks) in rural mountain landscapes. |
| **ProGIreg*** (Productive Green Infra-structure for post-industrial urban regeneration) www.progireg.eu | ProGIreg uses nature for urban regeneration with and for citizens. In the case study cities, using eight different NBS, the project will create productive green infrastructure that not only helps improve living conditions and reduces vulnerability to climate change, but also provides measurable economic benefits to citizens and entrepreneurs in post-industrial urban districts. |
| **European Sustainable Cities Platform**  http://www.sustainablecities.eu | This platform was launched in 2016 following the 8th European Conference on Sustainable Cities and Towns in the Basque Country. It focuses on the uptake of The Basque Declaration as the main outcome of the conference. The platform includes the Transformative Actions Database, which presents existing transformative actions in line with the Basque Declaration as good practice. |
| **Naturvation*** (NATure-based URban innoVATION) https://naturvation.eu | NATURVATION works in fields as diverse as urban development, innovation studies, geography, ecology, environmental assessment, and economics. The project assesses what NBS can achieve in cities, examines how innovation is taking place, and works with communities and stakeholders to develop the knowledge and tools required to realize the potential of urban NBS for meeting SDGs. |
| **NAIAD***  www.naiad2020.eu | NAIAD is aimed at operationalizing the insurance value of ecosystems for water-related risk mitigation by developing and testing NBS concepts, tools and applications on nine demo sites across Europe. At the core of the project is the physical and socio-economic analysis of demo sites that are supported with complex modelling and forecast activities which, in cooperation with the insurance sector, strive to propose NBS as a technically sound and financially viable option for local investors. |
| **Nature4Cities***  www.nature4cities.eu | Nature4Cities is creating a comprehensive reference Platform for NBS that offers technical solutions, methods and tools to empower urban planning decision making. This will help address the contemporary environmental, social and economic challenges that face European cities. |
| **Connecting Nature*** https://connectingnature.eu | The project is measuring the impact of the initiatives on climate change adaptation, health and well-being, social cohesion and sustainable economic development in cities. Innovative actions to foster the start-up and growth of commercial and social enterprises active in producing NBSs and their products is an integral part of the work. It forms a community of cities that fosters peer to peer learning and capacity building through co-development and co-operation between local governments, SME’s, academic research and community partners. It produces an NBS tool kit and guidebook for cities as well as an open database platform of 1490 NBS cases from urban Europe. |
| **URBAN Green-UP***  http://www.urbangreenup.eu | URBAN Green-UP aims at developing, applying and validating a methodology for Renaturing Urban Plans to mitigate the effects of climate change, improve air quality and water management and increase the sustainability of our cities through innovative NBSs. Its objective is the development, application and replication of Renaturing Urban Plans in a number of (non)European partner cities. |
| **Unalab** (Urban Nature Labs)*  https://www.unalab.eu | The UNaLab partner cities aim to address the challenges that cities around the world face today by focusing on climate and water-related issues within an innovative and citizen-driven paradigm. With three demonstration cities, seven replication cities, and several observers, it aims to develop smarter, more inclusive, resilient and increasingly sustainable societies through innovative NBS. |
| **GrowGreen*** (Green Cities for Climate and Water Resilience, Sustainable Economic Growth, Healthy Citizens and Environ-ments) www.growgreenproject.eu | GrowGreen aims to create climate- and water-resilient, healthy and livable cities by investing NBS. The main objectives are: 1) to contribute to the evidence base of NBS in cities for cost-effective, replicable means of increasing urban climate and water resilience, social, environmental and economic benefits, to underpin the development of NBS policies and the global NBS market; 2) to develop an easy-to-use and replicable approach to support the development and implementation of NBS strategies in cities; 3) to support the creation of the required conditions for the NBS implementation. |
| **Think Nature***  https://www.think-nature.eu/ | The objective of the project is the development of a platform that supports the understanding and promotion of NBS. The ThinkNature platform is an umbrella for all projects on NBS that are funded by the EU H2020 program. These projects include: Eklipse, Inspiration, NAIAD, Nature4Cities, Naturvation, Biodiversa, URBAN Green-UP, Unalab, GrowGreen and Connecting Nature. |
| **CLEVER Cities***  https://clevercities.eu/ | This project uses NBS to address urban challenges and promote social inclusion in cities across Europe, South America and China. Together, local governments, civil society, universities and businesses design, test and implement NBS for urban planning processes in several cities in Europe. Other cities replicate and tailor the NBS to their local needs. The aim is to increase and improve local knowledge of NBS, demonstrate their impact and benefits and develop the strategies. |
| **REGREEN***  https://www.regreen-project.eu/ | REGREEN substantially improves the evidence and tools for supporting co-creation of NBS in urban settings, implementation of decision support systems for planning and governance, and development of business models for realizing spatially relevant NBS that provide multiple ES and wellbeing. REGREEN will utilize advanced socio-spatial and land-use models, in combination with ecological expertise, to determine best-case solutions for re-greening selected cities through ULLs. |
| **ROBUST*** (Rural-Urban Outlooks: Unlocking Synergies)  http://rural-urban.eu/ | The goal of ROBUST is to a) advance our understanding of the interactions and dependencies between rural, peri-urban and urban areas, and b) to identify and promote policies, governance models and practices that foster mutually beneficial relations. Improved governance arrangements and synergies will contribute to Europe’s smart, sustainable and inclusive growth. |
| **ENABLE**-Enabling Green and Blue Infrastructure Potential in Complex Socio-ecological Regions www.nableprojectenable.eu | ENABLE explores which circumstances lead to the benefits of GBI being realized in cities. This project examines various benefits urban GBI can offer, how they are distributed, and who benefits from them. It aims to develop new methods and tools for maximizing the capacity of green and blue interventions in neighborhoods and metropolitan regions while considering local stakeholders’ perspectives. |
| **URBiNAT*** (Healthy corridors as drivers of social housing neighbor-hoods for co-creation of social, environmental and marketable NBS) https://urbinat.eu/ | This project focuses on the regeneration and integration of deprived social housing neighborhoods. It does so through an innovative and inclusive cataloguing of NBS that ensures sustainability and mobilizes driving forces for social cohesion. NBS focus on the public space to co-create with citizens’ new urban, social and nature-based relations within and between neighborhoods and co-plan a healthy corridor as an innovative and flexible NBS. |

**Table 2.** Classification of NBS presenting their main types included in the data and knowledge base (approach from [13, 22], with own additions)

| **Classification according to the scale or scope** | **Main aim of the interventions** | **Interventions included in the class** |
| --- | --- | --- |
| **Building-scale interventions** | Refurbishing pre-existing buildings, design of new buildings | - green roof (actions on rooftops) - green facades, green walls and vertical greening - balcony green - actions in community spaces of the buildings (e.g. rooftop farms, insect hotels, bee hives) - indoor vertical greenery (walls, ceilings, atrium) |
| **Interventions in public spaces** | Public space regeneration, urban land renewal, design of public living areas to improve greening, to increase social cohesion and integration | - large urban parks or forests - pocket parks - neighborhood green spaces - green corridors - botanical gardens |
| **Urban agriculture/farming** | Interventions to increase social cohesion and integration, contributing to awareness raising, networking, citizens engagement, sustainable living/consumptions | - community gardens - allotments - horticulture |
| **Interventions in water bodies and systems for water management** | Renaturing and recovery of river courses and wetlands, ponds, and lakes, NBS for water retention and flood prevention/mitigation | - renaturing rivers, canals, streams - restoration of ponds and lakes - sea cost interventions - wetland, bog, marsh - sustainable urban drainage system (SUDs) - rain gardens - swales, strips |
| **Interventions in linear transport infrastructures (grey-green interventions)** | Road projects, mobility plans, redevelopment, and greening streets – naturing actions for both high capacity (i.e., (a) roads-railways, etc. and (b) greening streets) | - alley and street trees - railroad bank and tracks - riverbank green - green parking lots |
| **Interventions in natural areas and land management** | Master plans to use/manage spaces, public space plans, green infrastructure strategies, agriculture, and forestry promotion plans | - natural protected areas - peri-urban parks - rural land management |
| **Ecological education and awareness raising-related interventions** | Raising awareness of environmental issues, stakeholder and citizen involvement, knowledge transfer | - ecological festivals - workshops - master classes and education activities |
| **Other types of greening** | Greening and renaturing interventions not included in the previous categories | - abandoned and derelict spaces - institutional green spaces - green playground/school grounds - house gardens |

**Table 3.** Classification scheme and key variables used for NBS data and knowledge base development

| **Category** | **Description** |
| --- | --- |
| **City size** | In terms of the analysis of the city sizes, the OECD (2012) classification of functional urban areas was used:  - XS (**extra small urban areas**) - urban settlements with a population of 2,500–50,000 people  - S **(small urban areas)** with a population of 50,000–100,000 people  - M **(medium sized urban areas)** with a population between 100,000 and 250,000 people  - L (**large sized urban areas)** with a population between 250,000 and 500,000  - XL (**metropolitan areas)** with a population between 500,000 and 1 million people  - XXL (**large metropolitan)** areas with a population between 1 million and 5 million people  - GS (**global city)**, areas with a population of 5 million people or more |
| **Phase** | finalized, ongoing, pilots ongoing, design progress (submitted) |
| **Sustainable development goals (SDG) addressed** | Based on the list of UN (2015), the relation to one or more of 17 SDG was indicated for each particular NBS (e.g. SDG3 – good health and well-being, SDG6 – clean water and sanitation, SDG8 – decent work & economic growth, SDG 10 – reduced inequalities, SDG 13 – climate action, SDG 14 – life below water, SDG 15 – life on land, etc.) |
| **Relation of the intervention to NBS** | - NBS  - partly NBS  - non-NBS (meaning that it is a project that deals with grey infrastructure, organization of event or policy document) |
| **NBS relation to climate change** | - yes  - no |
| **Specific aspects of climate impact** | - **Heat** **and drought** include the climate impacts on cities regarding urban heat islands, extreme temperatures and heat waves as well as droughts and water scarcity  - **Flooding** includes extreme precipitation, storm events, superficial flooding and sea level rise  - **Loss of biodiversity** indicates the extinction of species (plant or animal) or local reduction/loss of species in a certain habitat.  **- Air quality –** indicates that air is currently polluted or it is forecast to become polluted  - **Energy consumption** – include relevance to energy saving technologies and activities  - not relevant |
| **Project category** | - big data  - governance and planning  - changing mentalities  - workshop  - green infrastructure  - water management  - grey infrastructure  - citizen engagement  - citizen and stakeholder engagement |
| **Financing source** | - Public local authority’s budget  - Public national budget  - EU funds  - Funds provided by NGOs  - Crowd-sourcing  - Private foundations  - Corporate investment |
| **Budget** | The total projected costs/monetary resources of an NBS intervention (in euro) |
| **Primary beneficiaries** | - public  - private  - public-private  - governmental institutions  - scientific or technical advisors |
| **Method of implementation** | The methods were classified as:  - **Change in physical infrastructure** include projects that demonstrate different elements of built infrastructure (e.g. dams, levees, irrigation channels, piped drainage, impermeable surfaces) and deal with conventional grey infrastructure development.  - **Change in legislation and regulation** refers to projects that deal with policy trends and legislation that influences the implementation of NBS, issues/reforms needed in national legislation, policies, and procedures and local government regulations and decisions before it will be possible to build/implement the NBS.  - **Financial incentives** refers to projects that deal with the creation of financial incentives to support NBS for risk management, e.g. tax breaks, etc., or provide a structured overview of business model and financial arrangements for NBS).  - **Methods for the integration of ecosystem services (ES)** – projects on recognizing and integrating ES into policy making, planning and implementation of specific projects, Integrated Assessment and Valuation of ES.  - **Big data** include projects that are concentrating on the development of databases, big green data, or provide meta-analysis that includes green indicators to facilitate social innovation and new business opportunities, as well as help stakeholders use big data to make decisions about NBS.  **- Networking** refers to the projects that promote networking and cooperation among different stakeholders/actors involved in NBS or supported networking of existing initiatives in the field of urban sustainable development.  **- Sharing knowledge** refers to projects committed to collaboratively generating, managing and sharing green growth knowledge and data to mobilize a sustainable future. It also includes projects that contribute to the analyses and sharing of innovative solutions, share experiences and knowledge of urban GI.  **- Application of best practice methods** includes the projects that present evidence-based practices and methods or techniques that have documented outcomes and an ability to replicate as key factors.  **- Green infrastructure development** refers to the Green infrastructure planning to enhance implementation of NBS. It aims at developing networks of green and blue spaces in urban areas (green roofs, natural drainage systems, green infrastructure and biodiversity plan, pollution and waste management, etc.). |
| **Project scale** | The project scales were classified into six levels:  - **Continental** refers to Europe-wide projects.  **- National** refers to the project that include area of the whole country.  - **Regional** refers to projects that involved more than one municipality.  - **City** refers to projects that include multiple projects throughout a city or a project that operates in more than one district.  - **District**/**neighborhoods** refers to projects that affect multiple streets or distinct neighborhoods.  - **Site-specific projects** affect only the immediately surrounding area.  **- Building** – e.g. interventions such as rooftop greening, green walls or facades, etc. |
| **Motivations** | The classification of this category was adopted from Cater and Kazmierczak (2010) and modified:  - **Climate change adaptation and mitigation –** includes NBS intervention dealing with *response to current climate* (implicates a motivation based on prior or recent climate events or climate conditions), *adaptation to climate change* (refers to a motivation based on impending or prospective climate impacts) and *mitigation of climate change* (refers to the reduction of sources or enhancement of sinks of greenhouse gases).  - **Quality of life/human well-being/attractiveness of place** refers to physical and mental health improvements, enhancement and upgrading of public spaces, aspects of *recreation* that includes activities as well as the improvement of public spaces for activities and sports.  - **Nature conservation** refers to the enhancement of the ecological conditions and the prevention of biodiversity/ecosystem losses.  - **Water management** **and flood control** refers to projects that aim to meet the EU water directive or other water management related motivations.  - **Improved (green) planning** refers to green infrastructure planning that enhances the implementation of nature-based solutions and aims to develop networks of green and blue spaces in urban areas (green roofs, natural drainage systems, green infrastructure and biodiversity plan, pollution and waste management, etc.).  - **Awareness raising** includes NBS interventions that aim to help raise awareness for nature-based solutions (NBS).  - **Sharing/Producing new knowledge** refers to the projects committed to collaboratively generating, managing and sharing green growth knowledge and data to mobilize a sustainable future. It also includes projects that contribute to the analyses and sharing of innovative solutions, shared experiences and knowledge of urban GI.  - **Citizen engagement** includes NBS intervention that promotes citizen engagement and participation in policymaking and the design of NBS.  - **Citizen and stakeholder engagement** includes NBS interventions that promote stakeholder engagement throughout the whole NBS development process, from co-design of intervention to dissemination, in order to increase the societal impact of the funded research.  - **Networking** refers to projects that promote networking and cooperation among different stakeholders/actors involved in NBS or supported networking of existing initiatives in the field of urban sustainable development. |
| **Claimed additional benefits** | The additional benefits provided by NBS intervention are classified as the following:  - **Climate change adaptation and mitigation –** include NBS intervention dealing with *response to current climate* (implicates a motivation based on prior or recent climate events or climate conditions), *adaptation to climate change* (refers to a motivation based on impending or prospective climate impacts) and *mitigation of climate change* (refers to the reduction of sources or enhancement of sinks of greenhouse gases).  - **Quality of life/human well-being/attractiveness of place** refers to physical and mental health improvements, enhancement and upgrading of public spaces, aspects of *recreation* that include activities as well as the improvement of public spaces for activities and sports. *Tourism* refers to improvements and the creation of tourist attractions and landmarks.  - **Improved (green) planning** refers to green infrastructure planning that enhances the implementation of nature-based solutions and aims at developing networks of green and blue spaces in urban areas (green roofs, natural drainage systems, green infrastructure and biodiversity plan, pollution and waste management, etc.).  - **Awareness raising** include NBS interventions that aim to help raise awareness for nature-based solutions (NBS).  - **Sharing/Producing new knowledge** refers to the projects committed to collaboratively generating, managing and sharing green growth knowledge and data to mobilize a sustainable future. It also includes projects that contribute to the analyses and sharing of innovative solutions and shared experiences and knowledge of urban GI.  - **Citizen engagement** includes NBS intervention that promote citizen engagement and participation in policymaking and the design of NBS.  - **Citizen and stakeholder engagement** includes NBS interventions that promote stakeholder engagement throughout the whole NBS development process, from co-design of intervention to dissemination, in order to increase the societal impact of the funded research.  - **Networking** refers to the projects that promote networking and cooperation among different stakeholders/actors involved in NBS or who support the networking of existing initiatives in the field of urban sustainable development.  - **Big data** includes projects that concentrate on the development of database, big green data or provide meta-analysis that includes green indicators to facilitate social innovation, new business opportunities, as well as help stakeholders use big data to make decisions about NBS.  **- Sustainable living** describes a lifestyle that attempts to reduce an individual's or society's use of the natural resources, and one's personal resources, in order to reduce the ecological footprint by altering the methods of transportation, energy consumption, and/or diet (e.g. following the overall principles of sustainable development).  **- Social inclusion** includes projects that show how NBS help to increase social contact, provide inclusion and feeling of belonging; improved self-esteem, confidence and mood; and an increase in personal achievement through meaningful activity.  **- No additional benefits measurable**. |
| **Initiator of NBS** | This presents an information on governance arrangements, including initiated bodies:  **- public, private, public-private, governmental institutions, scientific or technical advisors** |
| **Stakeholders** | Shows key actors involved in the planning and implementation of NBS:  **- public, private, public-private, governmental institutions, scientific or technical advisors** |
| **Management set-up/Governance** | Indicates governance mechanisms for the implementation and stewardship of NBS:  **- Co-governance or hybrid governance** (mix of responsibilities between government and non-government actors)  **- Government-led** (variety of governmental bodies – City Council and its departments, state authorities)  **- Led by non-government actors** (NGO, scientific or technical advisers, private company or citizen groups) |
| **Participatory approaches** | Relates to community involvement:  **Co-planning, Co-development, Co-implementation, Dissemination of information and education, Consultation (e.g. workshop, surveys), Citizen science**, etc. |

**Reference**

[22] L. Gutiérrez, G. García, I. García, Nature-Based Solutions for Local Climate Adaptation in the Basque Country: Methodological Guide For Their Identification and Mapping, Donostia/San Sebastián Case Study. Environmental Management Agency, Ministry of the Environment, Territorial Planning and Housing—Basque Government; Ihobe, Bilbao, Spain, 2017.
